# Simple SNP-based minimal marker genotyping for *Humulus lupulus* L. identification and variety validation

**DOI:** 10.1186/s13104-015-1492-2

**Published:** 2015-10-06

**Authors:** John A. Henning, Jamie Coggins, Matthew Peterson

**Affiliations:** USDA-ARS, 3450 SW Campus Way, Corvallis, OR 97331 USA; ROY FARMS, INC., 401 Walters Road, Moxee, WA 98936 USA; CGRB, ALS Building, Oregon State University, Corvallis, OR 97331 USA

**Keywords:** Genotyping, DNA fingerprint, Hop, Humulus, Minimal marker, Minor allele frequency, SNP, TASSEL, Variety identification

## Abstract

**Background:**

Hop is an economically important crop for the Pacific Northwest USA as well as other regions of the world. It is a perennial crop with rhizomatous or clonal propagation system for varietal distribution. A big concern for growers as well as brewers is variety purity and questions are regularly posed to public agencies concerning the availability of genotype testing. Current means for genotyping are based upon 25 microsatellites that provides relatively accurate genotyping but cannot always differentiate sister-lines. In addition, numerous PCR runs (25) are required to complete this process and only a few laboratories exist that perform this service. A genotyping protocol based upon SNPs would enable rapid accurate genotyping that can be assayed at any laboratory facility set up for SNP-based genotyping. The results of this study arose from a larger project designed for whole genome association studies upon the USDA-ARS hop germplasm collection consisting of approximately 116 distinct hop varieties and germplasm (female lines) from around the world.

**Results:**

The original dataset that arose from partial sequencing of 121 genotypes resulted in the identification of 374,829 SNPs using TASSEL-UNEAK pipeline. After filtering out genotypes with more than 50 % missing data (5 genotypes) and SNP markers with more than 20 % missing data, 32,206 highly filtered SNP markers across 116 genotypes were identified and considered for this study. Minor allele frequency (MAF) was calculated for each SNP and ranked according to the most informative to least informative. Only those markers without missing data across genotypes as well as 60 % or less heterozygous gamete calls were considered for further analysis. Genetic distances among individuals in the study were calculated using the marker with the highest MAF value, then by using a combination of the two markers with highest MAF values and so on. This process was reiterated until a set of markers was identified that allowed for all genotypes in the study to be genetically differentiated from each other. Next, we compared genetic matrices calculated from the minimal marker sets [(Table 2; 6-, 7-, 8-, 10- and 12-marker set matrices] and that of a matrix calculated from a set of markers with no missing data across all 116 samples (1006 SNP markers). The minimum number of markers required to meet both specifications was a set of 7-markers (Table 3). These seven SNPs were then aligned with a genome assembly, and DNA sequence both upstream and downstream were used to identify primer sequences that can be used to develop seven amplicons for high resolution melting curve PCR detection or other SNP-based PCR detection methods.

**Conclusions:**

This study identifies a set of 7 SNP markers that may prove useful for the identification and validation of hop varieties and accessions. Variety validation of unknown samples assumes that the variety under question has been included a priori in a discovery panel. These results are based upon in silica studies and markers need to be validated using different SNP marker technology upon a differential set of hop genotypes. The marker sequence data and suggested primer sets provide potential means to fingerprint hop varieties in most genetic laboratories utilizing SNP-marker technology.

**Electronic supplementary material:**

The online version of this article (doi:10.1186/s13104-015-1492-2) contains supplementary material, which is available to authorized users.

## Background

Hop is an important cash crop for the Pacific Northwest USA as well as several European countries, China, Australia, South Africa and other minor production regions. It is primarily used as a flavoring and bittering additive in beer brewing but alternative uses have become increasingly important [1, 2]. Hop is a dioecious perennial plant species propagated via rhizomatous cuttings. The female inflorescence (or hop “cone”) is the harvested product. While male hop plants are required for breeding purposes female hop plants will produce cones without pollination [3]. Male hop plants disperse pollen via air and if present near production hop yards, can pollinate and produce seed on female hop varieties. Seedlings from these crosses can supersede the previous genotype if they possess superior fitness. Furthermore, when new varieties are produced on yards previously producing a different variety, it is possible for escapes to continue production. Both of these scenario’s can be compounded over the life of a new hop variety planting with the result that a yard becomes contaminated. If rhizome cuttings are subsequently sold from this yard, the recipient grower could end up with either a partially or fully contaminated yard. Hop sales from this field are then rejected due to unexpected flavors or bittering capacity. In addition, farms located across the USA with historical importance have requested help in identifying feral hops growing on their property ([4]; Personal Observation). In these cases, the goal would be to eliminate the possibility that the unknown line is a currently available hop variety.

Regardless of the scenario, the hop industry does not currently have an efficient, accurate and widely available method for marker-based genotyping of hop accessions. Current means used by the National Clean Plant Network for genotyping hop are based upon 25 microsatellites that provides relatively accurate genotyping but cannot always differentiate sister-lines (Dr. Ken Eastwell, Personal Communication 2015). Patzak and Matoušek [5] reported on the use of expressed sequence tagged, simple sequence repeat (EST-SSR) markers as a means of differentiating hop varieties. The reported PCR-based method utilized 30 EST-SSR markers to differentiate 11 different hop genotypes representing a wide genetic pool. Unfortunately, no broad-based evaluation of related and unrelated genotypes was reported. In addition, a significant number of PCR steps (30) are required to utilize this method. Koelling et al. [6] reported on the identification of a 952 new SSR markers identified from expressed sequence tagged data sets deposited with National Center for Biotechnology Information (NCBI: http://www.ncbi.nlm.nih.gov/). These 952 markers were tested across 8 different cultivars to determine differentiation power of the markers. The combination of all 952 markers was successful in differentiating among the 8 cultivars. Again, no minimal number of SSR markers was identified in this study. Howard et al. [7] reported on the genotyping capabilities of diversity array technology markers (DArT) in hop. While Howard et al. [7] demonstrated DArT markers as having sufficient capability to resolve closely related hop genotypes, its cost and dependence upon a single service provider (Diversity Array Technology Inc.; http://www.diversityarrays.com/) limit availability. What is needed is a simple, widely available methodology that utilizes a minimal number of markers to differentiate between both related and unrelated hop genotypes.

Single nucleotide polymorphic (SNP) markers represent the most abundant source of variation that can be utilized to differentiate among genotypes especially as they are found in both coding [8] and non-coding regions [9]. Recent genome sequencing work (data not published) shows the presence of a SNP every 346 bp on average in hop. Matthews et al. [10] was the first group to identify and report on next generation sequencing derived SNP markers having identified 17,128 SNPs. This group utilized SNP markers to genotype hop varieties and concluded that a highly filtered group of 3068 SNP markers resulted in a dendrogram that did not significantly differ from dendrograms obtained using the lower stringency filtered set of 16,106 SNP markers. However, no minimum number of markers required to differentiate among all genotypes were identified and reported.

The minimal number of markers chosen for DNA fingerprinting cultivars has been examined in numerous crops (see [11] for review) and computer programs have been written to address this application [12] across any plant species. In essence, the primary means of identifying the minimal number of markers consists of some means of ranking markers upon their effectiveness at describing population variation and reiteratively including more and more markers until all genotypes in the population can be genetically differentiated. This process was utilized to identify a small set of SNP markers that could, upon validation, be utilized to differentiate among genetically diverse hop accessions and be widely adaptable and available to genetic laboratories worldwide.

## Results and discussion

A total of 374,829 SNP markers were identified using the TASSEL-UNEAK Ver 3.0 pipeline [13] across a population of 121 individual varieties and germplasm accessions. Filtering of SNP sites, as well as filtering out individuals with poor sequencing results, was accomplished using TASSEL ver 4.3.4 [14] resulting in a set of 32,206 high quality SNP markers across 116 genotypes (Table 1). SNP marker filtration settings were set to require presence in 80 % of all genotypes for acceptance into the data set. Presence of greater than 50 % of all 32,206 SNP markers was set as cut-off for inclusion of a variety into the final data set. Some genetic lines with higher than 50 % missing gamete calls were kept in the study due to their importance in hop production (Hallertau Mittelfrueh, Wye Zenith, etc., Table 1). Cut-off specifications did not differ significantly from those utilized by Matthews et al. [10].

**Table 1:** Summary genotypic information of the results of partial sequencing for 116 varieties and experimental lines

Taxa name	Number of sites	Proportion missing gametes	Proportion heterozygous gametes
19105x19058M	32,206	0.28507	0.18871
21397X19058M	32,206	0.18466	0.18729
21397x21381M	32,206	0.15677	0.17053
21521x64035M	32,206	0.18596	0.13964
21534x21088M	32,206	0.4392	0.10476
21534x64037M	32,206	0.52431	0.09608
61021x21618M	32,206	0.21002	0.21445
Ahil	32,206	0.03049	0.36273
Alliance	32,206	0.03732	0.21691
Alpha Aroma (AlphAroma)	32,206	0.39319	0.17024
Apolon	32,206	0.05154	0.28246
Aquila	32,206	0.1772	0.27239
Atlas	32,206	0.11662	0.28633
Aurora	32,206	0.14354	0.18298
Backa	32,206	0.02468	0.29732
Banner	32,206	0.1142	0.32253
Bianca	32,206	0.14423	0.22318
Blisk	32,206	0.08713	0.36031
Bobek	32,206	0.17009	0.16915
Brewers Gold (BrewGold)	32,206	0.28507	0.26866
Buket	32,206	0.05192	0.21582
Bullion10A	32,206	0.40048	0.22913
Canadian Red Vine (CanadRV)	32,206	0.27271	0.23507
Canterbury Golding (CantGold)	32,206	0.14643	0.15526
Cascade	32,206	0.3255	0.22064
Cekin	32,206	0.18571	0.17781
Celeia	32,206	0.05766	0.26788
Centennial	32,206	0.17133	0.24194
Cerera	32,206	0.13302	0.22355
Chinook	32,206	0.1383	0.26647
Columbia	32,206	0.33441	0.15395
Comet	32,206	0.18922	0.25911
Crystal	32,206	0.10868	0.30513
Dunav	32,206	0.23157	0.13835
Early Prolific (E_Prolific)	32,206	0.06421	0.21249
Early Promise (E_Promise)	32,206	0.03006	0.21634
East Kent Golding (EKentGold)	32,206	0.06483	0.21535
Eastern Gold (EastGold)	32,206	0.22095	0.18928
Eastern Green (E_Green)	32,206	0.16379	0.17467
Eastwell Golding (EastGolding)	32,206	0.29116	0.11792
English Inter. 30 (EnglishInt30)	32,206	0.3341	0.0996
Eroica	32,206	0.11917	0.26562
FirstChoice	32,206	0.14323	0.22448
FuggleH	32,206	0.12516	0.18949
FuggleN	32,206	0.0857	0.2075
FuranoAce	32,206	0.12808	0.21036
Galena	32,206	0.38692	0.26569
Hallertau Gold (Hgold)	32,206	0.33096	0.11496
Hallertau Magnum (Hmagnum)	32,206	0.25899	0.17465
Hallertau Mittelfrueh (Mittelfrueh)	32,206	0.67742	0.0463
Hallertau Tradition (Htradition)	32,206	0.17407	0.14282
Hersbrucker Alpha (HersbrAlpha)	32,206	0.48696	0.07656
Hersbrucker Pure (HersbPure)	32,206	0.29786	0.12347
Hersbrucker Red Stem(HersbrRedSt)	32,206	0.06629	0.2267
Hersbrucker6	32,206	0.12982	0.17527
Hersbrucker8	32,206	0.04707	0.24249
Horizon	32,206	0.29004	0.1965
Hueller Bitter (HuelBitter)	32,206	0.03521	0.37111
Hybrid_2	32,206	0.39747	0.14934
Keyworths Early (KeywEarly)	32,206	0.17602	0.18427
Keyworths Midseason (KeyMidseas)	32,206	0.50528	0.15967
KirinC-601	32,206	0.25194	0.21036
KirinII	32,206	0.22378	0.26565
Kitamidori	32,206	0.18096	0.21097
Liberty	32,206	0.16286	0.16932
Lublin	32,206	0.60681	0.0597
Magnumx21267M	32,206	0.20984	0.21381
Mt.Rainier	32,206	0.12457	0.24576
Nadwislanka	32,206	0.08095	0.22092
Neoplanta	32,206	0.21996	0.15608
New Zealand Hallertau (NZHaller)	32,206	0.15227	0.23691
Newport	32,206	0.09275	0.24251
Northern Brewer (N_Brewer)	32,206	0.21956	0.15174
Nugget	32,206	0.27486	0.15633
Olympic	32,206	0.13122	0.29321
Omega	32,206	0.03704	0.22652
Orion	32,206	0.10194	0.18165
Perle	32,206	0.07421	0.20046
Pride of Kent (Pride_Kent)	32,206	0.26327	0.19063
Pride of Ringwood (PrideRing)	32,206	0.37487	0.15845
Saazer clone (Osvald72Y)	32,206	0.05564	0.26113
Saazer38	32,206	0.13563	0.18299
Santiam	32,206	0.06334	0.35109
Savinja Golding (SavGolding)	32,206	0.2352	0.13686
Saxon	32,206	0.07893	0.21433
Scarlet	32,206	0.19711	0.21966
Shinshuwase	32,206	0.19844	0.30134
SorachiAce	32,206	0.1492	0.20448
Southern Brewer (S_Brewer)	32,206	0.07319	0.25495
Spalter Select (SpaltSelect)	32,206	0.19931	0.1367
Sterling	32,206	0.14547	0.17772
Stricklebract (Strickle)	32,206	0.17292	0.2563
Styrian	32,206	0.26334	0.13467
Sunbeam	32,206	0.23052	0.20951
Sunshine	32,206	0.03322	0.23503
SuperAlpha	32,206	0.0866	0.31166
Talisman	32,206	0.28066	0.23918
Tardif de Bourgogne (Tardif)	32,206	0.11808	0.18833
Teamaker	32,206	0.31466	0.20682
Teamakerx19046M (Teax19046M)	32,206	0.34202	0.17163
Teamakerx21119M (Teax21119M)	32,206	0.25222	0.22697
Tettnanger	32,206	0.29926	0.12606
Tolhurst	32,206	0.07222	0.20669
Toyomidori	32,206	0.16177	0.22822
Ultra	32,206	0.17248	0.1974
USDA21734	32,206	0.17264	0.25824
Vojvodina (Vojvod)	32,206	0.02742	0.28998
Whitsbred Golding (WhitGold)	32,206	0.05415	0.22438
Willamette	32,206	0.43871	0.18969
Wuerttenburger (Wuertt)	32,206	0.29178	0.12613
Wye Challenger	32,206	0.27296	0.12466
Wye Target	32,206	0.23701	0.19757
Wye Viking	32,206	0.09039	0.19696
Wye Yeoman	32,206	0.25638	0.14648
Wye Zenith	32,206	0.411	0.06146
Yugoslavia Golding (YugoGold)	32,206	0.15783	0.17273

**Fig. 1:**
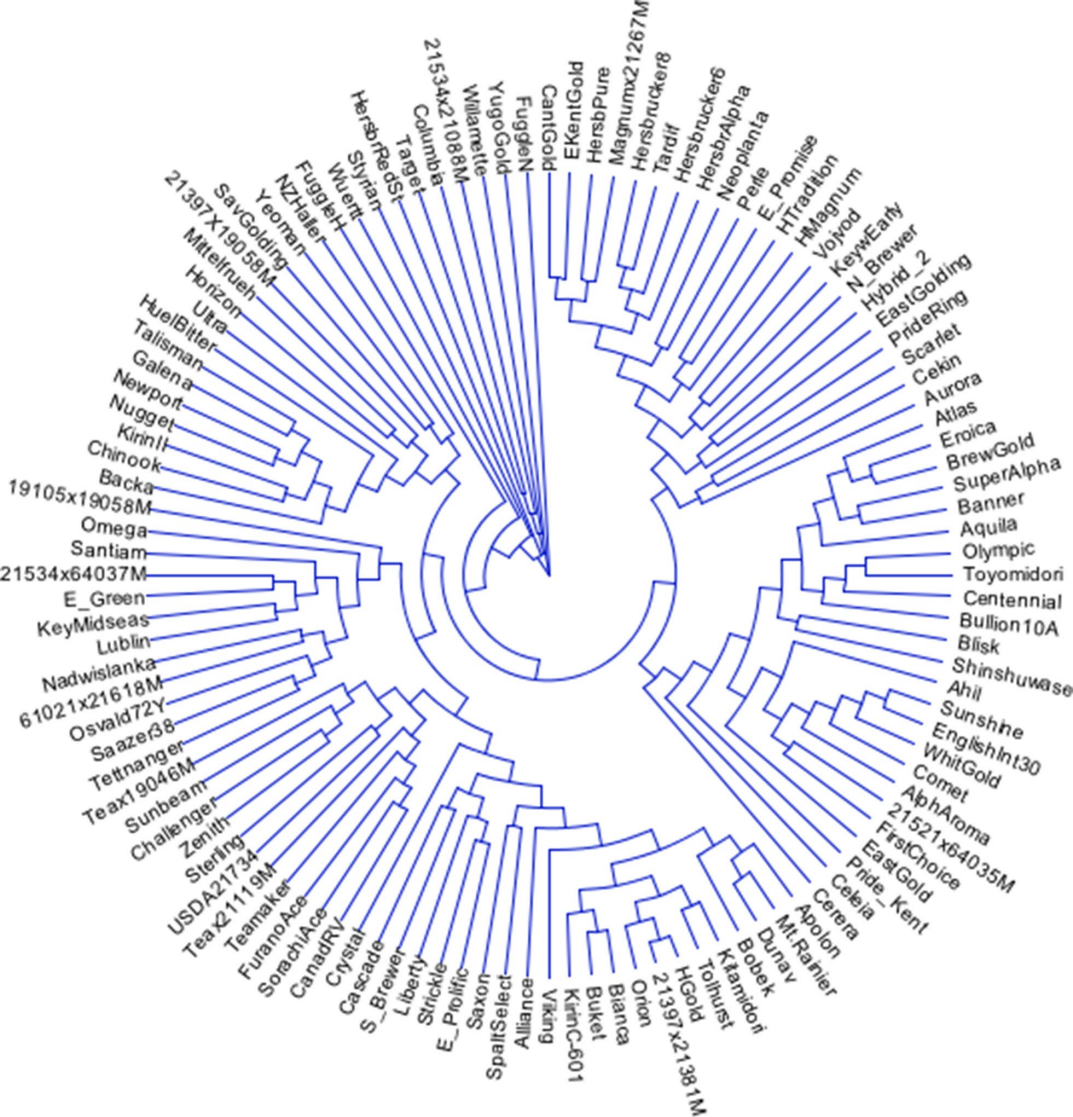
Dendrogram of the 116 hop varieties and germplasm resources as determined using the seven SNPs proposed as the minimal number of markers to genetically differentiate hop accessions

**Fig. 2:**
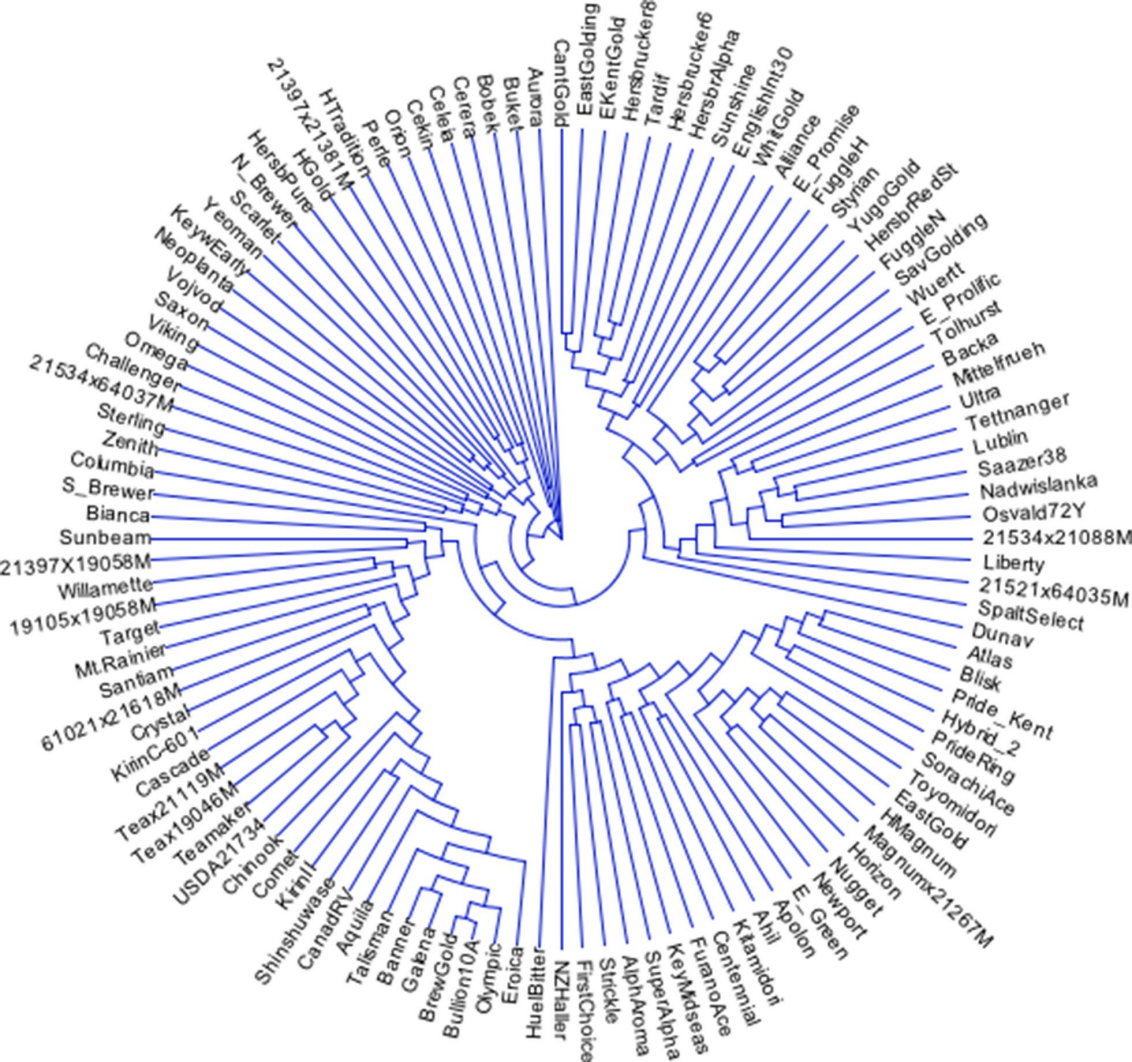
Dendrogram of hop 116 hop varieties and germplasm resources as determined by use of 1006 SNP markers with no missing data out of the pool of 32,206 SNPs utilized for this study

Labels for lines present in dendrogram (Figs. 1, 2) are defined in the first column (Taxa Name)

Genotype summaries using all 32,206 SNP markers were obtained using TASSEL. Included in TASSEL’s genotype summary were estimations of the minor allele frequency (MAF). MAF-values are important statistics utilized to filter out markers with high error potential (MAF <0.05) or provide the best discrimination power between genotypes [15]. Ranking of MAF-values from highest to lowest identified numerous markers with MAF <0.5. SNP markers that were heterozygous across all genotypes were discarded from consideration. Using a reiterative process of additive inclusion of a single marker with highest MAF values we identified a set of six (6) SNPs that were capable of differentiating among all 116 genotypes in the study.

The dendrogram resulting from the use of these six SNP markers did not match up well with dendrograms developed from the use of a complete set of SNP markers (data not shown). As a result, we continued to include additional markers with high-MAF values to the minimal set of markers and then compared the resulting genetic diversity matrices to a matrix calculated from a complete set of 1006 markers (no missing markers from data set) (Table 2). It was determined that the seven SNP markers (Table 3; Fig. 1) with highest MAF-values were required to both differentiate all 116 genotypes and define statistically similar dendrograms (approximate Mantel *T* test; t = −15.7471, p = 0.00001) as compared to a complete set of 1006 SNP markers (Fig. 2).

**Table 2:** 3-way Mantel’s t test [23] for cophenetic comparisons among genetic distance matrices comparing genetic distances calculated via 6-, 7-, 8-, 10-, and 12-markers (X-matrix, no missing markers) to that of a matrix calculated with 1006 SNP markers (Y-matrix, no missing markers) using a Z-matrix calculated from 32,217 markers (20 % missing marker data allowed)

#Markers	Mantel’s		
	r	t test	p
6	−0.0010	−0.1125	0.4552
7	−0.2190	−15.7471	0.0000
8	−0.2217	−15.9771	0.0000
10	−0.2105	−13.0887	0.0000
12	−0.2199	−13.5649	0.0000

This test uses residuals of regression of X on Z and of Y on Z

**Table 3:** List of seven SNP sequences (SNP shown in parentheses) differentiating all 116 hop accessions

>TP137094
agaaaattcatatttgggaatgtatatgaatgattacatargagggaacccacatttggattttaacatgttgtctccac aattttgtgggcatgatcagcagccttactcgactgctacttcaatattggaaatggatggtgcaattgtaactact(a/g)ct taaatgccacaattcccatcatcgtcatcatcatgtgctgctatgaaaagtaatggtgcaatgggacatatcgattatca taacacataatgcatacatgaaat
>TP15403
tcaggacaagtgcttatagatggtgttgatttgaagaatttgcagctcaaatggataagggagaagattggactagttag ccaagaacctgttctgtttgcagc(a/c)actttaagagaaaatatagcttatgggaaggaaaatgcaacagatgaggagatta aaacagccattgagcttgctaatgctgctaaattcattaacaaacttcctcaggtaaacacagaaaaaacccatctcttt gtttcaagttatgtacttttcttc
>TP245055
agagttctgtggttgcacacgtagaggattcccttcttgctgttttgaggatcatttgttttccaatgggtgcctccttt gaccgttaagtcaacgccagctgcaatgcc(c/t)agaggggttcctatctggaaaggaaggaaccccactgagattcgaaata tggctagaatgactcccaagggaagccataggaacatgacaagagtcgcagccggtgtgggcaagaaggctagtctccca tcatggaaaataagtggtttgggg
>TP295074
aaacgacccctaaactttaagcacccgtgcaccatcgagtaccctccactgtcacggcccaaactaagctcttgaatcac tttagacggggttgggtcggctgccac(a/g)tgagcttgcaagttcggaatagaaaggagtgttggagtggatatggctgcca agggcatcagctgctttattttcaaggccaggtcggtacacaatgtaaaaatcgtagcccaataacttggtgagccattt ttgatgtttt
>TP400349
ccaaaatcatcaagcaactcgactcacccgccgccagagaacaagccatgcgcaccatcatattccagtccgacgcacgc gccgcccaccctgttggtggctgctacca(c/t)atcatccaagaactcctgcgcaagattgaagctaccaaagctgaactcga cctcgttaatcaagatctcgccgtctaccgtgctgccgccgcggccgctgcagtgccaccacaacctcagggtgtctctt ctcatcatcatgtggatgatcatc
>TP411590
agcgacaaatttctgaacatcatccctcattcctgaccaatgcaaatctcttgcaagtcgctgaaatgttttgaaaaccc ctgaatgaccctcaatgttgttgctatggtattcttggagtaacagtggaataaa(c/t)ggtgaagaagagggaataaccaaa cgaaccctaaactttaggcacccatgcaccatcgagtaccctccaccgtcaagacccaaactaagmttttgaatcacttt agacagggttggatcrgtggccac
>TP437202
gctctagaaggaacaagatgccatttcccttcaccatacttgtctatgcaatccctaagaagatcgtcttcttctctggt ccatgcgcctttcctcaccgctgct(g/c)tcccagacgaaccgccctcagtttgttccgttactagtactgtcaccatattaa tattgatattgctgcgcataatgtatttatatgaattatgtaaaatacgatatataatataatatgngaatactganaag ntaattaactagctttccagtcct

PCR-based methodology to screen SNP markers varies from simple (single strand conformational polymorphism, SSCP; [4] to resequencing using next generation sequencing. This study identified a set of SNP markers that could potentially be used to differentiate hop genotypes. We propose the use of high-resolution melting (HRM) curve analyses as a simple and rapid means to perform genetic fingerprinting on hop genotypes. Utilizing a draft hop genome, we aligned the raw reads for informative SNP markers to extend reads to a total length of 264-bp. Primer3 software identified optimum primer sequences that can be used to develop Amplicons for HRM analysis (Table 4).

**Table 4:** Suggested primers pair sets and amplicon specifications for high resolution melting curve analysis of seven SNP markers differentiating among 116 hop genotypes

SNP marker	Direction	Length	Primer sequence	Pair Tm		Product Tm
				Diff	Size	
TP137094	Forward primer	24	GGGCATGATCAGCAGCCTTACTCG	2.39	99	59.71
TP137094	Reverse primer	25	TGACGATGATGGGAATTGTGGCATT	2.39	99	57.32
TP15403	Forward primer	24	TGCAGCTCAAATGGATAAGGGAGA	0.54	114	55.84
TP15403	Reverse primer	25	CCTCATCTGTTGCATTTTCCTTCCC	0.54	114	56.38
TP245055	Forward primer	20	TGGGTGCCTCCTTTGACCGT	0.64	83	57.89
TP245055	Reverse primer	23	TCAGTGGGGTTCCTTCCTTTCCA	0.64	83	57.26
TP295074	Forward primer	19	AGACGGGGTTGGGTCGGCT	0.1	92	59.81
TP295074	Reverse primer	21	AGCAGCTGATGCCCTTGGCAG	0.1	92	59.71
TP400349	Forward primer	19	GCCGCCCACCCTGTTGGTG	2.26	86	60.55
TP400349	Reverse primer	23	ACGAGGTCGAGTTCAGCTTTGGT	2.26	86	58.28
TP411590	Forward primer	27	AATGACCCTCAATGTTGTTGCTATGGT	0.15	110	57.18
TP411590	Reverse primer	23	GATGGTGCATGGGTGCCTAAAGT	0.15	110	57.33
TP437202	Forward primer	24	CTTCTTCTCTGGTCCATGCGCCTT	0.37	70	59
TP437202	Reverse primer	21	ACGGAACAAACTGAGGGCGGT	0.37	70	58.63

Several of the accessions used in this study are thought to be clonal selections from other lines contained in this study. As an example, Savinja Golding is thought to be a clonal selection from Fuggle (see: “Slovenian Styrian Goldings: https://bsgcraftbrewing.com/slovenian-styrian-goldings) as are Fuggle H and Fuggle N (A. Haunold, Personal Communication, 2014). In addition, Hersbrucker 6 and 8 are thought to be clonal selections from the original German ‘Hersbrucker’ landrace (see: USDA ACCESSION No. 21514; http://www.ars.usda.gov/SP2UserFiles/person/2450/hopcultivars/21514.html). All these “clonal selections” show sufficient phenotypic differences from the related lines as well as parent lines to suggest genetic differences between them, although differences are expected to be minor. The inclusion of clonal selections was to determine if a sufficiently robust method could be devised to differentiate among such lines.

Previous work in hop have focused upon the identification of male plants from a population of offspring [16] or genetic diversity and DNA fingerprinting using older marker technology such as STS, SSR, AFLP, RAPD and DArT [7, 17–19]. In all publications, differentiation of accessions required the full compliment of markers used for defining genetic diversity in hop populations. In several reports, a few hop varieties were not differentiated from one another and complete validation was not possible given the marker technology used. Furthermore, none of the published reports identified a subset of markers that could be used independently to fingerprint hop varieties.

In this study, use of the full compliment of 1006 SNP markers found in all cultivars (Fig. 2) and use of the minimum number of markers (7 SNPs—Fig. 1) completely differentiated all female lines contained in this study. In this report, 7 SNPs were identified that effectively differentiated all varieties and accessions present in the study. The hop lines chosen for this study represent a broad spectrum of hop lines from around the world. Some of the varieties evaluated in this study were not adequately differentiated using older marker technology such as AFLP or SSR’s. Thus, these older technologies have sufficient limitations in their usefulness for variety validation or identification. Partial sequencing through next generation sequencing technology allows for the identification of thousands of SNP markers from across the genome. These markers are not limited to clustered regions such as SSRs and DArT markers [16, 20] and are therefore more representative of the genome. Because of their distribution throughout the genome, SNP markers offer a greater likelihood of differentiating among accessions.

The 7 SNPs identified in this study were the minimum number of markers required to differentiate all the hop accessions in this study. They have not yet been tested using high resolution melting (HRM) or other SNP detection methods. Furthermore, the use of these 7 SNPs as a discriminating tool for samples consisting of mixtures of different cultivars has not been tested but may have limited applicability given the small number of markers used. The primers for use in HRM are reported for implementation by other projects (Table 3). If one or two of these SNPs prove to be insufficient for use in HRM or other PCR techniques, there are additional SNP markers that can be utilized (Supplementary Data).

## Conclusions

This note reports on the identification of a minimal number of markers (7 SNPs) required to differentiate among 116 widely divergent hop accessions including clonal selections and sister hop lines. As such, it is the first publication outlining a simple widely available protocol for the identification of, and discrimination among, hop varieties. The SNPs and associated primer sequences for HRM analysis are provided and supplementary data provided to aid genetic.

Laboratories ensure their own set of markers that can be used for differentiation among hop lines.

## Methods

Plant material consisted of 121 genotypes (varieties and experimental germplasm) contained in the USDA-ARS hop genetics and breeding program located at Corvallis, OR. Due to poor DNA quality of a few of the lines, the final sample number used was 116. DNA was extracted using DNAeasy Kits (Qiagen Inc) with the exception that the amount of RNase A was doubled and the QIAshredder spin column was not used. Library preparation, sequencing and were as reported by Elshire [21]. Because hop does not currently have a reference genome, SNP identification and production of hapmap files were accomplished using the TASSEL-UNEAK pipeline (http://www.maizegenetics.net/tassel/docs/TasselPipelineUNEAK.pdf). Resulting hapmap was analyzed by TASSEL 5.2.1 [14]. Marker and genotype summaries were exported as csv-format files which were imported into Microsoft^**®**^ Excel^**®**^ for Mac 2011. Minor allele frequency (MAF) were calculated in TASSEL and subsequently sorted from highest to lowest values. Initially, the top two markers with the highest MAF values were chosen for data analysis. These two markers with the highest MAF values were filtered into a separate data file in TASSEL v 5.0 using the “filter sites” option and genetic diversity values estimated from this filtered data. The resulting genetic diversity matrix was scanned for presence of genetic diversity estimates equal to zero. If present, the process was repeated adding the next marker with highest MAF value. These steps were reiterated until all genetic diversity estimates were greater than zero (matrix with six SNP markers having the highest MAF values). Additional high-MAF, SNP markers were added to this set of six SNPs to form additional genetic distance matrices (genetic distance matrices formed from 7-, 8-, 10- and 12-markers) for comparison to a complete set of polymorphic markers with no missing data (1006 SNP markers). NTSYSpc V2.21c [22] was used to estimate correlations between genetic matrices for minimal marker sets (6-, 7-, 8-, 10-, 12-markers) and the complete data set using 3-way Mantel’s t test [23] and a matrix calculated (constant or “Z-matrix”) from the original set of 32,206 SNP markers.

The 64-bp reads representing minimal marker data sets were aligned with a USDA-ARS/OSU draft hop genome (http://hopbase.cgrb.oregonstate.edu/app_dev.php/) to extend reads by 100-bp on either side of the 64-bp read using Geneious Pro ver 5.5.9 (http://www.geneious.com, [24] (Table 3). As an aid to interested parties, we developed primer pairs (Table 4) that are appropriate for high-resolution melting curve analyses [25] using Primer3 [26]. Default settings were used and product size was limited to a range of 70- to 115-bp length. Other PCR-based SNP assays are available and can be designed using the information in Table 3.

## Availability of supporting data

The data set supporting the results of this article is included within the article while the hapmap file from which this study derives is included as supplementary files (Additional file 1).

## Additional file

10.1186/s13104-015-1492-2 Supplementary data file consisting of the hapmap flat file of the 32,206 SNP markers used to identify the minimal set of seven SNP markers

## Abbreviations

AFLP: amplified fragment length polymorphism
DArT: diversity array technology
HRM: high resolution melting
MAF: minor allele frequency
RAPD: restriction amplified polymorphism DNA
SNP: single nucleotide polymorphism
SSR: single sequence repeat
STS: sequence tagged site
